# Role of legs and foot adhesion in salticid spiders jumping from smooth surfaces

**DOI:** 10.1007/s00359-021-01466-6

**Published:** 2021-03-10

**Authors:** Hanns Hagen Goetzke, Walter Federle

**Affiliations:** Department of Zoology, University of Cambridge, Cambridge, UK

**Keywords:** Jumping spiders, Jump kinematics, Locomotion, Foot contact, Adhesion

## Abstract

Many spiders and insects can perform rapid jumps from smooth plant surfaces. Here, we investigate how jumping spiders (*Pseudeuophrys lanigera* and *Sitticus pubescens*) avoid slipping when accelerating. Both species differed in the relative contribution of leg pairs to the jump. *P. lanigera* accelerated mainly with their long third legs, whereas their short fourth legs detached earlier. In contrast, *S. pubescens* accelerated mainly with their long fourth legs, and their short third legs detached earlier. Because of the different orientation (fourth-leg tip pointing backward, third-leg tip pointing forward), the fourth-leg tarsus pushed, whereas the third-leg tarsus pulled. High-speed video recordings showed that pushing and pulling was achieved by different attachment structures. In *P. lanigera*, third-leg feet made surface contact with setae on their distal or lateral claw tuft, whereas fourth-leg feet engaged the proximal claw tuft, and the distal tuft was raised off the ground. *S. pubescens* showed the same division of labour between proximal and distal claw tuft for pushing and pulling, but the claw tuft contact lasted longer and was more visible in the fourth than in the third legs. Experimental ablation of claw tufts caused accelerating spiders to slip, confirming that adhesion is essential for jumps from smooth substrates.

**Supplementary Information:**

The online version contains supplementary material available at 10.1007/s00359-021-01466-6.

## Introduction

Many arthropod species are able to jump to catch prey, to escape from predators, or to move in challenging terrains. In a forward jump, large forces have to be applied parallel to the ground to achieve high accelerations. On rough surfaces, tarsal spines or cuticle can interlock with sufficiently large surface asperities, but jumping from smooth surfaces is likely more difficult: if forces are only produced by classic friction, the take-off angle in a jump would be limited by the friction coefficient μ (Amontons’ law of friction: ${F}_{\parallel }=\mu {F}_{\perp }$, where ${F}_{\parallel }$ is the force parallel to the surface and ${F}_{\perp }$ is the force normal to the surface). Values of μ between rigid and dry surfaces are typically smaller than 1; for example the friction coefficient of beetle cuticle on glass is given as μ = 0.35 (Dai et al. 2002). With a friction coefficient of μ = 0.35 between the foot and the substrate, arthropods would only be able to perform steep upward jumps (the limiting take-off angle is $\alpha ={\mathrm{tan}}^{-1}\left({F}_{\perp }/{F}_{\parallel }\right)={\mathrm{tan}}^{-1}\left(1/\mu \right)\cong 70^\circ$), which would be very inefficient. In fact, most arthropods perform jumps with take-off angles significantly lower than 70°. How do they achieve this?

Many climbing arthropods have evolved soft pads on their feet, allowing them to climb even on smooth vertical and inverted surfaces. Typically, these adhesive structures are arrays of microscopic hairs or smooth pads found on the tarsus and pretarsus (Beutel and Gorb 2001; Scherge and Gorb 2001). Most of the adhesive pads studied so far are shear-sensitive and only stick when the leg is pulled towards the body but detach when it is pushed away from it, thereby allowing rapid attachment and detachment on smooth surfaces (Bullock et al. 2008; Autumn and Puthoff 2016; Federle and Labonte 2019). Can these climbing pads also be used for jumps? Most jumping insects accelerate for the jump by pushing their hind legs away from the body, but the legs move in the pulling direction during detachment (take-off). Therefore, the loading of hind legs before and during take-off goes against the usual direction-dependence of adhesive pads: the legs are expected slip when the insect is attempting to accelerate, and the feet should stick when the insect is about to take off. This problem becomes even more challenging when arthropods jump from vertical or inverted take-off positions since they have to use their adhesive pads before the jump. Hence, as classic friction is insufficient and adhesive pads have the wrong direction-dependence, how are arthropods able to jump from smooth surfaces?

Here, we investigate whether and how jumping spiders (Salticidae) are able to jump from smooth surfaces and to what extent their attachment devices are specialized for jumping. Do spiders jump by pushing with their legs, and if so, how are their pads able to withstand high pushing forces and detach when pulled during detachment?

Jumping spiders are diurnal hunters known for their acute vision; they perform accurate jumps to move to target positions when they escape from predators or navigate through vegetation, or they jump to capture moving prey (Parry and Brown 1959a; Hill 2010a, b). Salticids are able to move easily across the smooth surfaces of plants, including leaf undersides and vertical stems. They possess well-developed claw tufts with adhesive setae on their pretarsus explaining their excellent ability to move rapidly on vegetation (Hill 1977, 2010c). As far as is known to date, spiders have no extensor muscles in the hinge-like femur-patella and the tibia-metatarsus joints (Shultz 1989, 1991). Instead, they generate hydraulic pressure by compressing the prosomato rapidly extend their legs (Parry and Brown 1959a, b). While jumps of wandering spiders (*Cupiennius salei*, Ctenidae) can involve several leg pairs (Weihmann et al. 2010), jumping spiders (Salticidae) use their third and fourth legs to jump (Ehlers 1939; Hill 2018). Some jumping spider species accelerate mainly with their third legs, whereas other species accelerate mainly with their fourth legs, and some species with both leg pairs (Ehlers 1939; Hill 2018). The relative contribution of each leg is likely determined by their length, as longer legs can contribute more to the jump than shorter legs. In this study, we investigate two species representing the two extremes—*Pseudeuophrys lanigera* which accelerates mainly with their third legs, and *Sitticus pubescens* which accelerates mainly with their fourth legs. We investigated for these two species whether and how they are able to jump from smooth surfaces, and whether they have developed any specific adaptations of their attachment structures.

## Materials and methods

### Study species

We collected 33 *Pseudeuophrys lanigera* (Simon 1871; weight: 4.1 ± 1.4 mg) and 34 *Sitticus pubescens* (Fabricius 1775; weight: 9.6 ± 4.8 mg) jumping spiders of both sexes from houses and walls in Cambridge, UK. Spiders were kept in clear acrylic boxes (140 × 79 × 60 mm), and water was supplied via wet clay pebbles in a small tray; spiders were fed with fruit flies once per week.

### Morphology

To measure the length of the third and fourth legs, legs of adult spiders were cut off at the proximal end of the trochanter and placed flat on a coverslip. A photo of the leg was taken with a Canon EOS 60D digital camera attached to a Leica MZ16 stereomicroscope. The leg length was determined by measuring the length of each individual segment. The length of the claw tuft was measured in side view as the distance between the tips of the most proximal and the most distal setae.

Images of the pretarsi of five *P. lanigera* and nine *S. pubescens* were recorded using scanning electron microscopy (SEM). Spiders were anaesthetized, and their third and fourth legs mounted on SEM stubs, and freeze-dried at  – 20 °C over silica crystals for at least 2 weeks. Before imaging, spiders were transferred into a desiccator to warm up to room temperature and then sputter-coated with gold for 3 min (40 mA current) using an Emitech K550X sputter coater. Images were recorded with a Zeiss EVO LS10 SEM at 15 kV and with a FEI XL 30-FEG SEM at 10 kV.

### Jump performance on smooth surfaces

Jumps were recorded with a system of two synchronized high speed cameras, either at 500 and 1000 frames per second (fps) using two HotShot PCI 1280 cameras (NAC image technology, Simi Valley, CA, USA), or at 4700 fps using two Phantom cameras (Phantom V7.1 and V7.3, Vision Research, Wayne, NJ, USA). For each camera system, camera 1 was attached to an inverted microscope (Leica DM IRE2) to film from below the foot surface contact and movements during a jump from a glass coverslip using a 5 × lens and bright field epi-illumination from a 100 W mercury arc lamp. This illumination produces high-contrast images of adhesive contact areas (Federle and Endlein 2004). In most of the recordings, the foot of only one third or fourth leg was visible. Because of their small size (200–300 nm wide; Peattie and Full 2007), individual spatulae in contact were below the resolution limit (~ 4 µm) of the high-speed video recordings; nevertheless, setae of the claw tuft of which the spatulae were in close surface contact were distinctly darker and their contact areas could be approximately quantified using a thresholding algorithm in MATLAB. Because of the invisibility of the spatula contact, and the small size of individual seta contacts (< 10 µm), the error of the seta contact area measurements is probably large. Seta contact area was measured throughout the acceleration phase (defined as the period from the first visible movement of third or fourth legs to take-off) of each jump in which close contact of the claw tufts occurred. We also used interference reflection microscopy to image claw tufts in contact with a higher resolution, using a Leica DMR-HC upright microscope with a 100×/1.25 oil objective and monochromatic (546 nm) illumination (Peattie et al. 2011). Camera 2 filmed the jump from the side using a Nikon 105 mm macro lens; the camera was tilted downward, by a small defined angle β, to achieve an unobstructed view of the tarsi (Fig. S1). The two cameras were oriented at right angle to each other, so that the x-axis corresponded to the same direction in both views. From the jump’s azimuth angle ϑ recorded by camera 1, and the spider’s projected take-off angle γ recorded by camera 2, the true take-off angle α was calculated as $\alpha = \tan^{ - 1} \left( {\cos \left( {\vartheta - 90^\circ } \right)\tan \gamma /\cos \beta } \right)$. Spiders were placed on the glass coverslip and gently directed into the field of view of the inverted microscope with a fine paintbrush; however, recorded jumps where the paintbrush touched the spider during the acceleration phase were excluded from the analysis. A dead *Drosophila melanogaster* glued to a human hair was suspended as a lure 1.5 cm from the edge of the coverslip at the same height above a paper landing platform. To attract the jumping spider’s attention and motivate prey-catching jumps, the hair was touched to make the fruit fly on the hair move.

To manipulate adhesive structures, *S. pubescens* spiders were immobilized and mounted to expose individual legs. To study the individual performance of adhesive pads and determine their relative importance for jumping, individual claw tufts were ablated using micro scissors or damaged using a fine soldering iron tip.

In total, *n* = 97 jumps of *N* = 14 *P. lanigera* and *n* = 121 jumps of *N* = 18 *S. pubescens* jumping spiders were recorded. For spiders with intact claw tufts, only jumps directed towards the fruit fly were analyzed for both species. Additional jumps that were not directed at the target (48 jumps of *P. lanigera* and 96 jumps of *S. pubescens*) were not included in this analysis. For spiders with manipulated claw tufts, all jumps were analyzed. All data are reported as mean ± sample standard deviation. There was no significant difference in the kinematics, as well as leg and tarsus morphology between male and female spiders; we therefore pooled the data from both sexes. In statistical tests comparing third and fourth legs, individual jumps from the same animals are treated as independent jumps.

## Results

### Jump kinematics and contact to smooth surfaces in jumps of *Pseudeuophrys lanigera*

**Fig. 1:**
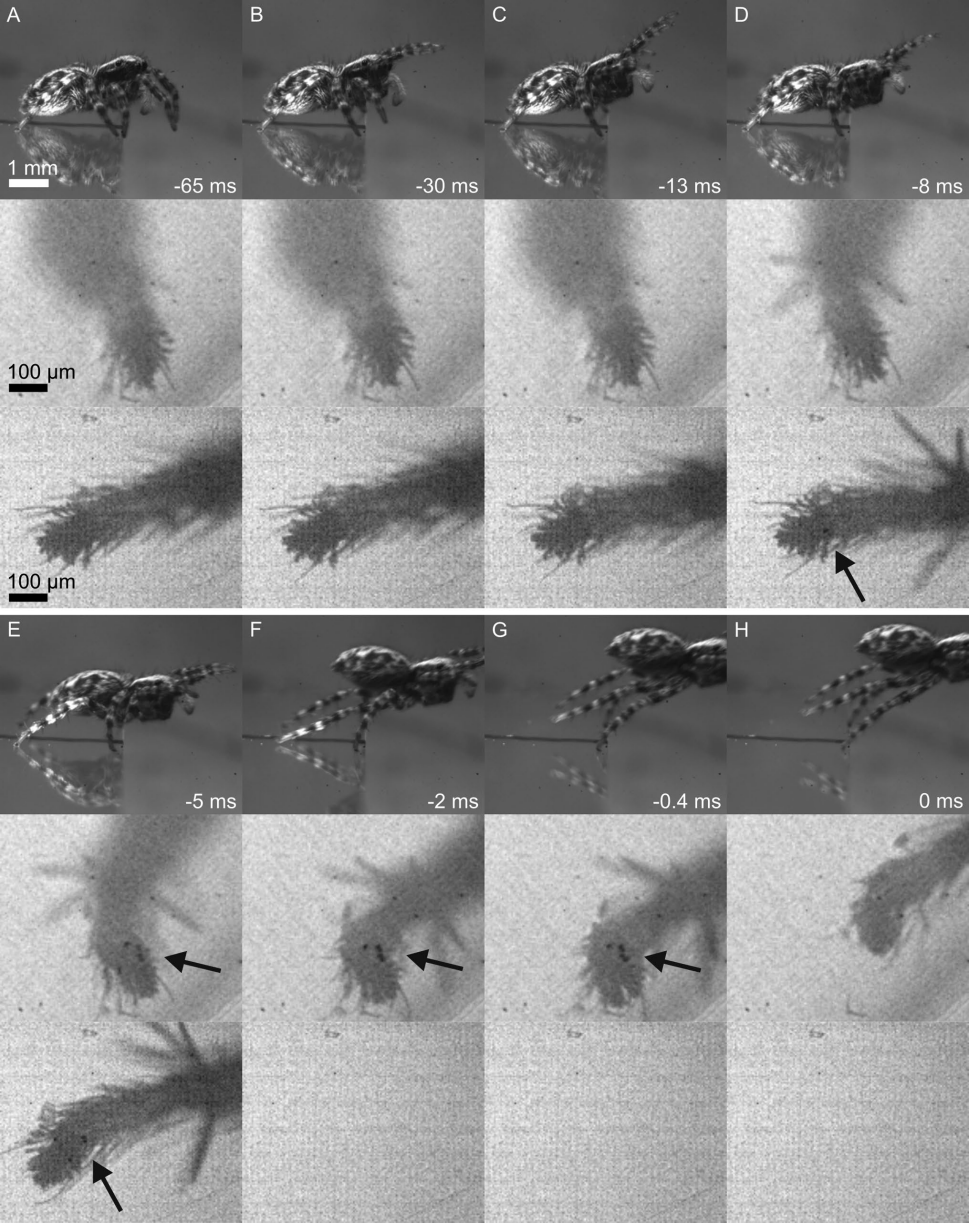
Jump of *Pseudeuophrys lanigera*; side view in the top row, right third leg in the middle row, right fourth leg in the bottom row (horizontal axis is the same in all three views; third leg recordings from a different jump but at equivalent times of the sequence); arrows point at contact areas. **a** Initial jump position: fourth leg’s tarsus points backward, third leg’s tarsus points laterally forward. **b** First leg pair lifted. **c** Second leg pair lifted. **d** Start of acceleration phase; large spines on the distal metatarsus are erected, and a small contact area is visible at the base of the claw tuft of the fourth leg. **e** Fourth leg’s pretarsus is pushed, so that the contact area shifts proximally relative to the foot; third leg’s pretarsus is pulled transversely, and close contact of setae becomes visible on the lateral side of the claw tuft, facing the jump direction. **f** Fourth leg’s pretarsus loses contact with surface, and third leg rotates around the foot contact. **g** Third leg’s pretarsus begins to detach. **h** Take-off

Jumping spiders accelerate for the jump using their third and fourth legs. We recorded 49 successful jumps of 10 adult *P. lanigera* spiders onto a *Drosophila* target. After identifying the target, *P. lanigera* spiders usually walked to the edge of the coverslip and oriented themselves towards the target. At the start of the acceleration phase, the first leg pair was raised off the surface (Fig. 1, supplementary Video 1). *P. lanigera* spiders started the jump by lifting the first leg pair even further (start of visible movement 54.9 ± 20.9 ms before take-off), followed by the lifting of the second leg pair. The third and fourth legs start to move 12.0 ± 1.8 ms before take-off, thereby accelerating the body in the direction of the jump. We observed a mean take-off angle of 24 ± 11° and a take-off velocity of 0.52 ± 0.08 m/s (Table 1).

**Table 1:** Comparison of jump kinematics, timing and foot contact for jumps of *Pseudeuophrys lanigera* and *Sitticus pubescens* spiders. Data are shown as means ± standard deviation

	*P. lanigera*	*n* (jumps, spiders)	*S. pubescens*	*n* (jumps, spiders)	Statistical test of species difference
Take-off angle	24 ± 11°	46,10	23 ± 11°	23,14	*t*_44.9_ = 0.4, *p* = 0.689
Take-off velocity	0.52 ± 0.08 m/s	48,8	0.70 ± 0.12 m/s	22,12	*t*_29.6_ = 6.4, *p* < 0.001
Start of first-leg movement ^1)^	– 54.9 ± 20.9 ms	49,11	– 47.6 ± 20.6 ms	23,14	*t*_43.8_ = 1.4, *p* = 0.171
Start of third and fourth-leg movement ^1)^	– 12.0 ± 1.8 ms	49,11	– 11.2 ± 2.8 ms	23,14	*t*_30.8_ = 1.2, *p* = 0.240
Spine erection visible in third (fourth) legs ^1)^	– 8.2 ± 1.0 ms ( – 7.7 ± 0.8 ms)	29,8 (40,9)	– 7.3 ± 1.7 ms ( – 7.8 ± 1.4 ms)	18,9 (20,10)	*t*_23.3_ = 2.0, *p* = 0.060 (*t*_24.0_ = 0.2, *p* = 0.813)
Detachment time of third (fourth) legs ^1)^	0 ( – 4.1 ± 1.1 ms)	49,11 (49,11)	-2.7 ± 1.5 ms 0	23,14 (23,14)	
Angle of third (fourth) pretarsus at start^ 2)^	64 ± 12° (154 ± 11°)	13,4 (8,2)	76 ± 13° (165 ± 11°)	5,4 (9,6)	*t*_6.7_ = 1.9, *p* = 0.105 (*t*_14.5_ = 2.0, *p* = 0.063)
Angle of third (fourth) pretarsus at take-off ^2)^	41 ± 9° (142 ± 16°)	16,5 (8,2)	88 ± 14° (173 ± 12°)	5,4 (12,9)	*t*_5.2_ = 7.2, *p* < 0.001 (*t*_11.7_ = 4.7, *p* < 0.001)
Duration of contact in third (fourth) tarsi ^3)^	8.6 ± 2.6 ms (5.4 ± 1.9 ms)	23,6 (10,3)	7.7 ± 1.7 ms (7.9 ± 2.6 ms)	3,3 (13,9)	*t*_3.3_ = 0.8, *p* = 0.480 (*t*_21.0_ = 2.6, *p* = 0.018)
Peak contact area of claw tufts in third (fourth) legs ^3)^	203 ± 207 µm^2^ (83 ± 92µm^2^)	16,6 (7,2)	117 ± 177 µm^2^ (244 ± 141 µm^2^)	11,8 (13,10)	*t*_23.7_ = 1.2, *p* = 0.260 (*t*_17.2_ = 3.1, *p* = 0.007)
Time of contact area peak in third (fourth) legs ^3)^	– 3.3 ± 1.9 ms (-6.9 ± 2.4 ms)	13,4 (5,2)	-6.9 ± 2.9 ms (-2.6 ± 1.7 ms)	5,5 (13,10)	*t*_5.4_ = 2.5, *p* = 0.049 (*t*_5.5_ = 3.7, *p* = 0.012)

^1^Times defined in relation to take-off (0 ms)

^2^Angles defined in relation to jump direction (0°)

^3^Accelerating third legs did not make visible adhesive contact in 3 out of 16 jumps in *P. lanigera*, and in 6 out of 11 jumps in *S. pubescens*. For fourth legs, no adhesive contact area was visible in 2 out of 7 jumps in *P. lanigera*, but it was always visible for *S. pubescens*

When accelerating for the jump, the third and fourth legs had different positions (lateral and posterior, respectively), and we observed a large difference in the orientation of the pretarsus. While the foot tips of the fourth legs were pointing backwards, the foot tips of the third legs were pointing laterally and forward (Fig. 1). Therefore, when the third legs moved from a lateral-anterior to a posterior position by initially rotating the leg in the body-coxa joint, the pretarsi were *pulled* backward. The initial backward movement of the foot via the rotation of the legs at the coxa was followed by a full extension of the legs. In 3 jumps by 3 spiders where the third legs did not grip well at the start of the acceleration phase, some backward and inward (towards the body midline) slipping of the tarsus was observed before a contact area became visible (mean sliding direction 49 ± 7° inward relative to the opposite of the jump direction).

By contrast, the fourth legs *pushed* during the acceleration phase by extending the trochanter-femur and femur-tibia joints. The fourth legs did not always extend fully and were only in contact with the surface during the first part of the acceleration. Fourth-leg tarsi detached from the surface earlier, 4.1 ± 1.1 ms before the take-off by the third legs. Once the fourth legs had reached their maximum extension, the third legs extended by straightening the trochanter-femur and femur-tibia joints, thereby accelerating the spider further until take-off.

The pressure-driven hydraulic leg extension of the spiders’ legs was evident from the erection of large spines on the tibia and tarsus of both third and fourth legs. The spines on the third legs erected and spread out slightly earlier than those on the fourth legs (Tables 1 and 2).

**Table 2:** Statistical comparisons between third vs. fourth legs

	*P. lanigera*	*S. pubescens*
Spine erection visible*	*t*_28_ = 3.3, *p* = 0.002	*t*_13_ = 2.8, *p* = 0.015
Duration of contact	*t*_23.2_ = 3.8, *p* = 0.001	*t*_4.3_ = 0.2, *p* = 0.881
Maximum contact area of claw tufts	*t*_21.0_ = 1.9, *p* = 0.068	*t*_19.1_ = 1.9, *p* = 0.070
Time of contact area peak	*t*_6.1_ = 3.0, *p* = 0.023	*t*_5.0_ = 3.1, *p* = 0.027

*Paired *t* test

### *P. lanigera* use their adhesive setae during the acceleration phase

At the start of the acceleration phase, the pretarsi of the third legs were pointing laterally forward at an angle of 64 ± 12° (13 jumps by 4 animals) to the body midline, which corresponds to the direction of the jump (0°). The pretarsi of their fourth legs were pointing backward at an angle of 154 ± 11° (8 jumps by 2 animals). At take-off, the pretarsi of the third legs were more aligned with the direction of the jump (third legs: 41 ± 9°; 16 jumps by 5 animals; paired *t* test: *t*_12_ = 7.1, *p* < 0.001), whereas the pretarsi of the fourth legs were pointing slightly more laterally (142 ± 16°; 8 jumps by 2 animals; paired *t* test: *t*_7_ = 2.4, *p* = 0.045).

**Fig. 2:**
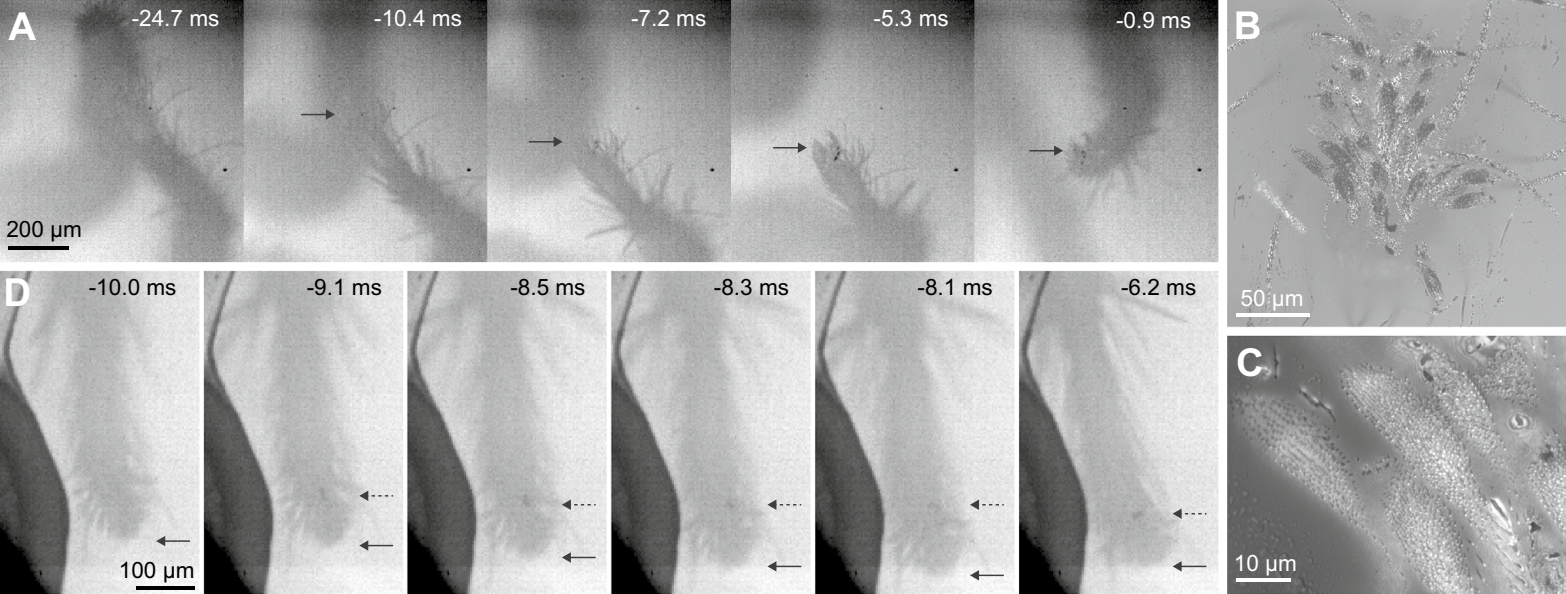
Sliding of the foot during jumps of *P. lanigera*. **a** Contact of third-leg pretarsus; example of a slide over a long distance. First image: before acceleration phase (note that the second leg partly obstructs the third-leg pretarsus). Seta contact becomes visible at  – 10.4 ms, and detachment occurs at  – 0.9 ms. Black arrows show the axial position of the small contact area. **b**, **c** Interference reflection microscopy images of third-leg pretarsus (**b**) and individual setae (**c**) in contact with glass substrate. **d** Position of the claw tuft contact area relative to the distal end of the fourth-leg pretarsus. The contact area becomes visible at the start of the acceleration phase at  – 9.1 ms; its position shifts proximally, away from the distal end of the tarsus (solid black arrow shows the position of the end of the tarsus; dashed arrow shows the axial position of the contact area); last image: before the fourth legs detach, the contact area remains stationary while the leg is pulled along the direction of the jump

During the acceleration phase, the pretarsi of the third legs were pulled backwards and a small number of setae came visibly into surface contact for 8.6 ± 2.6 ms (23 jumps by 6 animals), mostly on the side of the claw tuft facing the direction of the jump, corresponding to the distal or lateral side. For the third legs, the contact area of the claw tuft peaked at  – 3.3 ± 1.9 ms before take-off and reached 203 ± 207 µm^2^, whereas for the fourth legs even smaller contact areas of 83 ± 92µm^2^ were reached at  – 6.9 ± 2.4 ms before take-off. These contact areas became only visible during the acceleration phase; no adhesive contact was visible before. The third-leg claw tufts in contact sometimes slid backward over a short distance, giving an indication of the direction of the shear forces produced by them (mean sliding distance 36 ± 38 µm; mean sliding angle 178 ± 31° relative to the jump direction; 22 jumps by 6 animals; no sliding was observed in the other nine recordings; Fig. 2). At the end of the acceleration phase, the third legs rotated around the claw tuft in the direction of the jump (Fig. 1) and the contact area was mostly peeled off from the proximal to the distal side.

While the fourth legs pushed, a small contact area became visible at the proximal side of the claw tuft. These setae were in contact for 5.4 ± 1.9 ms (10 jumps by 3 animals). During the acceleration, the fourth pretarsus was loaded in the pushing direction, as was evident from the short backward sliding of the setae in contact (mean sliding distance 34 ± 20 µm; mean sliding angle 177 ± 22° relative to the jump direction; 34 ± 20 µm, 8 jumps by 3 animals). We also observed that during the push, the tarsus of the fourth leg moved even further backwards (distally) than its own contact area (by 22 ± 9 µm, 6 jumps by 1 animal; Fig. 2). This further confirms the pushing force; the displacement of the contact area relative to the tarsus may be explained both by the reorientation of setae on the proximal side of the claw tuft (from their default distal to a proximal orientation), and by the stretching of the claw tuft. This alignment of the setae with the force vector may allow the legs to transfer forces more efficiently in the pushing direction.

### Jump kinematics and contact to smooth surfaces in jumps of *Sitticus pubescens*, in comparison with *Pseudeuophrys lanigera*

We recorded 25 jumps of 13 adult *S. pubescens* spiders, under the same conditions as for *P. lanigera*; the kinematics of the jump, and the use of adhesive structures during the acceleration phase are compared with *P. lanigera* in Table 1.

**Fig. 3:**
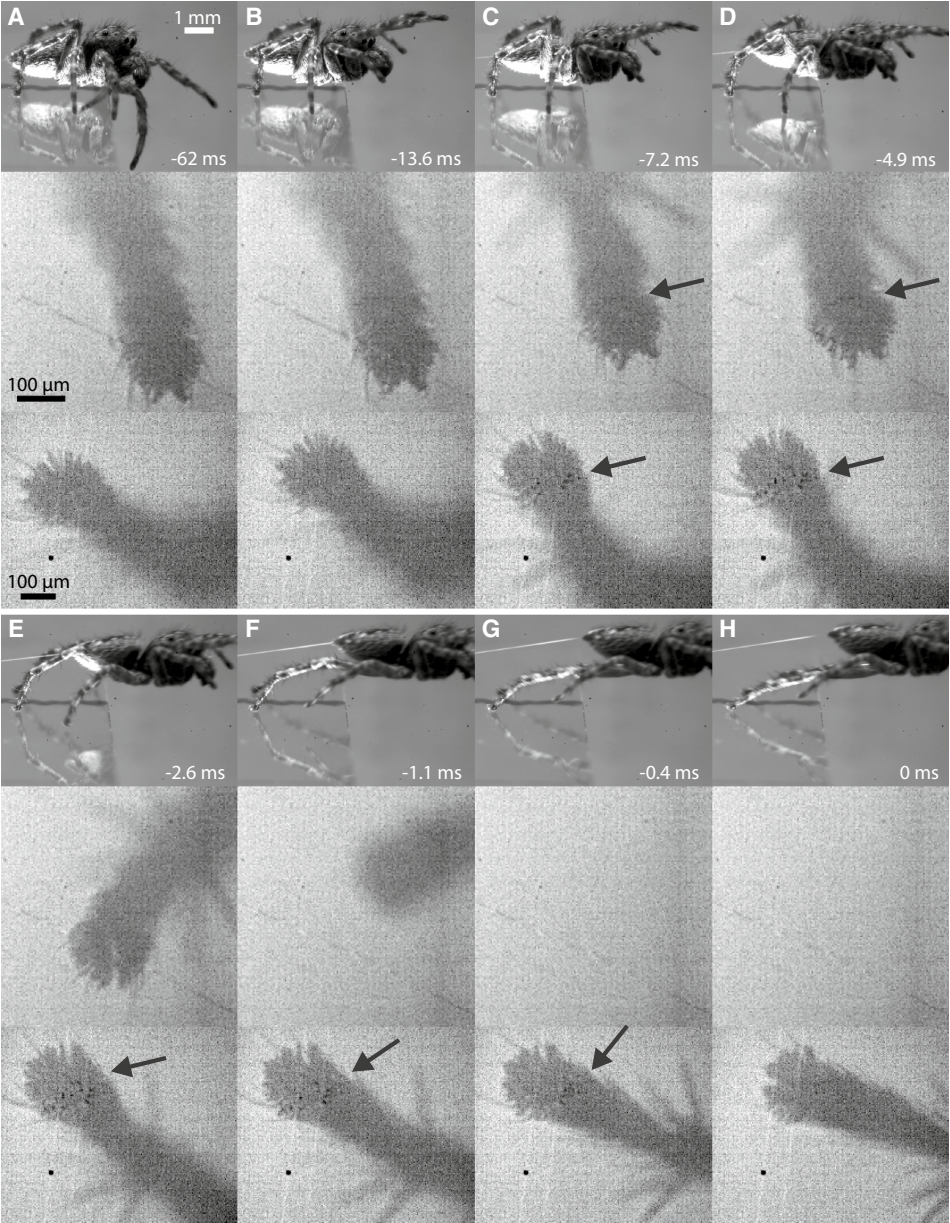
Jump of *Sitticus pubescens*; side view in the top row, right third leg in the middle row, right fourth leg in the bottom row (same horizontal axis in all three views; third-leg recordings from a different jump but at the equivalent times of the sequence), arrows point at contact areas. **a** Initial jump position: tarsus of fourth leg points backward, tarsus of third leg points laterally. **b** Start of the acceleration phase; first and second leg pairs are lifted. **c**, **d** Large leg spines are fully erected. Contact areas become visible on the proximal side of the claw tuft for the fourth leg pair. In the third leg, a small number of individual setae on the proximal side of the claw tuft can be seen in contact; in most other jumps, no contact was visible for the third legs. **e** Third leg detaches. **f** Left fourth leg detaches while the right fourth leg continues to extend; contact areas on the proximal claw tuft setae are still visible. **g** Contact areas of right fourth leg decrease while distal pretarsus is lifted off the surface. **h** Take-off

*S. pubescens* jumped with the same take-off angle but with a higher take-off velocity (0.70 ± 0.12 m/s, 22 jumps of 12 animals) than *P. lanigera*. In contrast to *P. lanigera*, *S. pubescens* spiders used mainly their long fourth legs during the acceleration phase while their shorter third legs were in contact only during the initial phase of the jump (Fig. 3, supplementary Video 2). The times when the different leg pairs started to move before the jump were similar in both spider species (*t*_43.8_ = 1.4, p = 0.171), and so was the timing of the erection of the leg spines in the third and fourth legs. However, clear differences were observed in the contact area, contact duration and detachment time of third and fourth legs, as well as in their orientation during the acceleration phase (Table 1). While the fourth legs detached on average 4.1 ms before the third legs in *P. lanigera*, the third legs did not always extend fully (further reducing their contribution to the jump) and detached 2.7 ms before the fourth legs in *S. pubescens*. The different relative contributions of third and fourth legs in both spider species were also reflected in a longer contact duration and larger contact area of the third legs in *P. lanigera*, but of the fourth legs in *S. pubescens* (Table 1). In *S. pubescens*, the foot tips of the third legs were oriented more laterally than in *P. lanigera* (*t*_6.3_ = 2.5, *p* = 0.043), whereas the pretarsi of the dominant fourth legs were pointing more backwards and were hence more aligned with the direction of the jump (*t*_7.0_ = 1.5, *p* = 0.178). In both spiders, there was a trend for the dominant leg pair to become even more aligned with the direction of the jump during the acceleration phase (angle of third legs reduced from 64° to 41° in *P. lanigera*, paired *t* test: *t*_12_ = 7.1, *p* < 0.001; angle of fourth legs slightly increased from 165 to 173° in *S. pubescens*, paired *t* test: *t*_8_ = 1.5, *p* = 0.178). By contrast, the subordinate legs became even less aligned during the acceleration phase (angle of fourth legs reduced from 154 to 142° in *P. lanigera*, paired *t* test: *t*_7_ = 2.4, *p* = 0.045; angle of third legs increased from 76 to 88° in *S. pubescens*, paired *t* test: *t*_4_ = 1.6, *p* = 0.196).

**Fig. 4:**
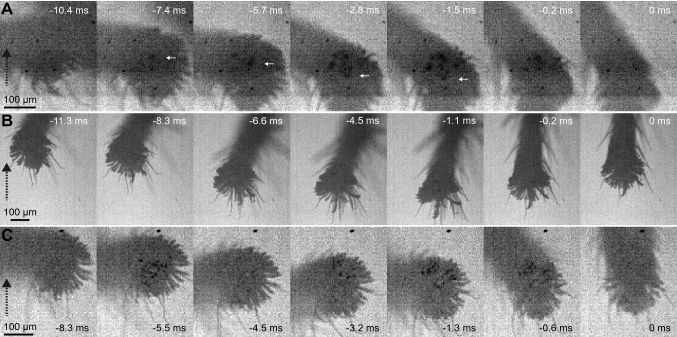
Sliding of fourth legs whilst in surface contact during the acceleration phase in *S. pubescens*. **a** Example of sliding of setae on the proximal side of the claw tuft. First image: before the acceleration phase; setae came into contact at  – 7.4 ms and detached at  – 0.2 ms as the claw tuft was raised at its distal end. White arrows show the y-position of one individual seta. **b** Fourth leg where very few setae in contact are visible; distal setae are reoriented during the push, exposing the claws further; at the end of the acceleration phase the tarsus is pulled along the direction of the jump and the distal setae return to their initial position. **c** Fourth leg where initially setae in the distal half of the claw tuft came into contact (second image), but detached or reoriented when pushed so that contact was almost lost (third image), but then setae on the proximal side of the claw tuft came into contact (fourth image). The direction of the jump is shown by a dashed black arrow

In the third legs of *S. pubescens*, a close surface contact of the claw tuft setae was only visible in 5 out of 11 jumps by 5 animals, and in 2 of these 5 jumps, the contact area was very small, with only a few setae in contact. In contrast, a close surface contact of claw tuft setae was more clearly visible in the fourth legs. During the acceleration phase, setae on the proximal side of the claw tuft were visibly in surface contact for 7.9 ± 2.6 ms (13 jumps by 10 animals); in some jumps, close contact of setae was initially visible on the distal side of the claw tuft but then other setae on the proximal side came into contact (Fig. 4).

We observed that during the acceleration phase, the fourth-leg claw tufts in surface contact sometimes slid backward and slightly outward over a short distance, giving an indication of the force direction (mean sliding distance 55 ± 23 µm; mean sliding angle 163 ± 8° relative to the jump direction; 7 out of 11 jumps by 6 animals, Fig. 4a). The pushing force produced via the claw tufts of the fourth legs is also confirmed by three jumps by two *S. pubescens* spiders, where setae on the distal claw tuft of fourth legs were in contact at the start of the acceleration phase. These setae moved proximally relative to the tarsus during the acceleration phase (Fig. 4b), or detached before setae on the proximal side of the claw tuft came into contact (Fig. 4c).

### Jumps with contaminated claw tufts, or experimental ablation of setae

We observed multiple unplanned instances of individual legs slipping on glass during the acceleration phase in both spider species; SEM inspection of one of the affected spiders showed that its claw tufts were contaminated with an amorphous substance that coated the microtrichia. In three jumps by three *P. lanigera* spiders, the spiders slipped with both third legs on the smooth glass surface and did not reach the target (see supplementary Video 3). These spiders failed to jump normally; they also slipped with their fourth legs and produced a forward (head-down) spin (12.5 ± 8.1 Hz); all three jumps failed to reach the target. In these jumps, the legs were nevertheless activated with the same order and timing as in successful jumps where the legs gripped (movement start of first legs: 37.0 ± 20.1 ms; third legs: 11.8 ± 1.4 ms; fourth legs fully extended 2.0 ± 1.4 ms before full extension of third legs). When only one of the third legs slipped (three jumps by three *P. lanigera* spiders), the spiders jumped sideways (towards the side of the slipped third leg) with a strong yaw, and again failed to reach their target.

We also studied the effect of claw tuft manipulations in *S. pubescens*. Once the claw tufts had been manipulated, the spiders could no longer grip with these legs during inverted locomotion on smooth surfaces. When the claw tuft setae of the third legs were ablated, the third legs slipped during the acceleration phase, but in contrast to *P. lanigera*, the spiders still managed to perform controlled jumps onto the target (13 jumps by 3 animals, supplementary Video 3). The slipping legs clearly showed the same timing and order of movements as in normal jumps, with an initial rotation of the leg in the body-coxa joint, followed by a full extension of the leg. When we ablated the claw tuft setae on individual fourth legs, the manipulated legs slipped backward at an angle of 167 ± 15° relative to the jump direction (21 jumps by 3 animals; supplementary Video 3), but the spiders still accelerated with the other fourth leg and managed to jump onto the target. However, these jumps had a significantly lower take-off velocity of 0.47 ± 0.09 m/s (12 jumps of 4 animals, paired *t* test: *t*_27.6_ = 6.3, *p* < 0.001).

### Leg and tarsus morphology

The third legs of *P. lanigera* are only slightly shorter than the fourth legs (third: 3.30 ± 0.21 mm, fourth: 3.57 ± 0.22 mm, *N* = 22). The ratio of third to fourth leg length is 0.92 ± 0.02. In comparison, the third legs of *S. pubescens* are much shorter than their fourth legs (third: 4.14 ± 0.34 mm, fourth: 5.95 ± 0.52 mm, *N* = 22), resulting in a much smaller leg length ratio of 0.69 ± 0.03 (difference between leg length ratio of *P. lanigera* and *S. pubescens*: *t*_41.5_ = 31.1, *p* < 0.001).

**Fig. 5:**
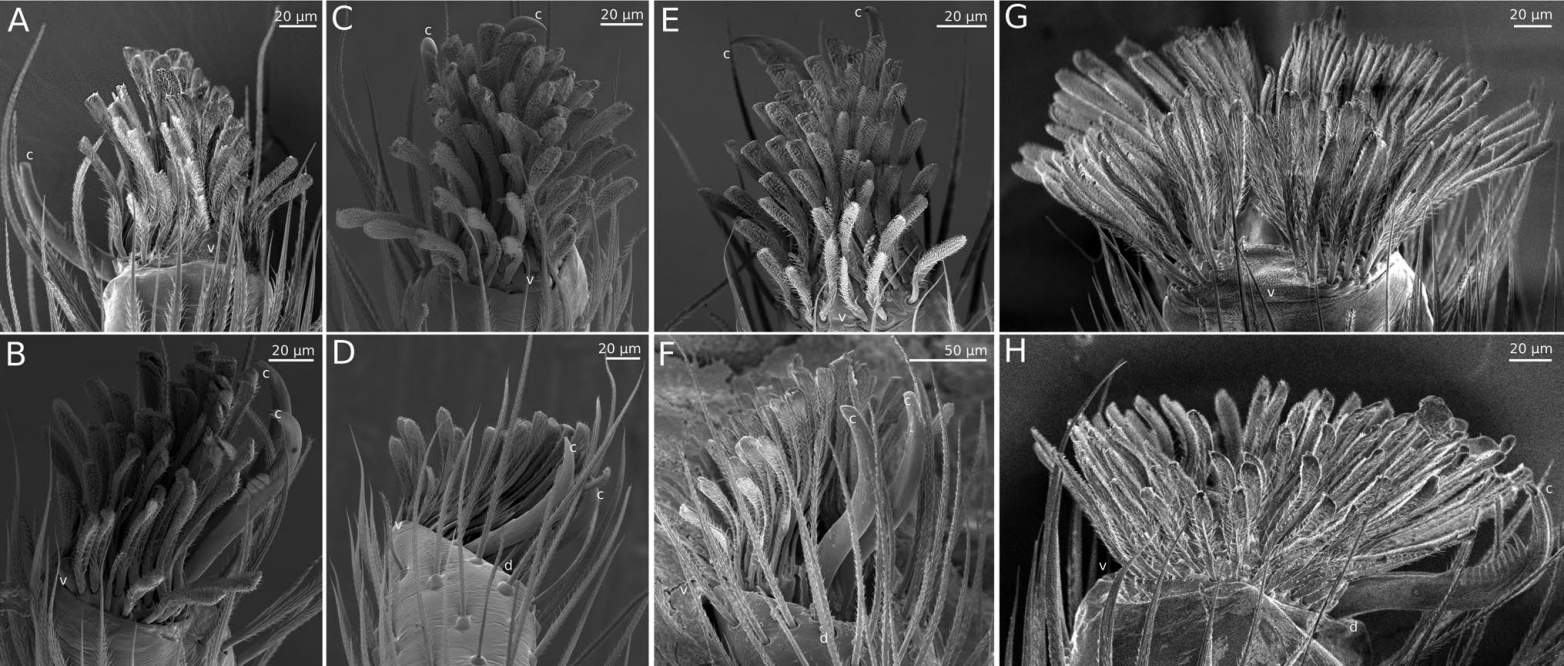
**a**–**d** SEM images of claw tufts of *P. lanigera*: third leg (**a**, **b**) and fourth leg (**c**, **d**); **e**–**h** SEM images of claw tufts of *S. pubescens*: third leg (**e**, **f**) and fourth leg (**g**, **h**). Top row: ventral views, bottom row: side views, c: claw tips, d: dorsal, v: ventral

For *P. lanigera*, the morphology of the pretarsus is very similar in the third and fourth legs (Fig. 5). Each claw tuft consists of two lobes covered by approximately 50 ± 4 (third legs) and 48 ± 7 (fourth legs) setae ranging in length from ~ 30 µm at the proximal end to ~ 100 µm at the distal end (estimated from four SEM images of third and four of fourth leg pretarsi of three animals). The flattened tips of the setae are between 7 and 12 µm wide. The ventral side of each seta is densely covered with microtrichia of up to ~ 5 µm length with flattened triangular tips (spatulae). The length of the third-leg claw tufts (measured in side view of the leg) was 143 ± 16 µm (21 animals), similar to the claw tufts on the fourth legs (140 ± 22 µm, 19 animals, paired *t* test: *t*_18_ = 0.3, *p* = 0.794).

The morphology of the third-leg claw tuft in *S. pubescens* is similar to that of *P. lanigera* but it is slightly longer and has more setae [Fig. 5, length 164 ± 20 µm (18 animals), 73 ± 25 setae (10 SEM images from 7 animals)]. The fourth-leg claw tuft of *S. pubescens* has a similar number of setae (73 ± 24, 6 SEM images from 4 animals), but it differs strongly on its proximal side from that of *P. lanigera*: in *S. pubescens*, the setae on the proximal side the claw tuft are oriented more ventrally and proximally (allowing them to point toward the body when the claw tuft is in surface contact) whereas the distal setae are oriented more dorsally and distally (Fig. 5h). This different orientation of the setae also leads to substantially longer fourth-leg claw tufts (227 ± 45 µm, 18 animals; difference to third legs: paired *t* test: *t*_15_ = 6.6, *p* < 0.001; difference to *P. lanigera*: *t*_24.4_ = 7.4, *p* < 0.001).

Neither in *P. lanigera* nor in *S. pubescens* did we observe any scopulae with adhesive setae on the ventral side of the tarsus. Interference reflection microcopy of claw tufts of live spiders in surface contact (Fig. 2b,c) showed that not only setae but also some feathery tarsal hairs came into contact with the surface in the periphery of the claw tuft (termed “pilosae” in Hill 2010c). However, the contact during the acceleration phase of the jumps was mainly made by the claw tuft setae.

## Discussion

Our findings show that *P. lanigera* and *S. pubescens* spiders are able to perform controlled jumps from smooth glass surfaces. They avoid slipping by bringing the claw tufts of their third and fourth legs into close surface contact during the acceleration phase. The mechanism of how the attachment of the claw tufts is achieved depends on the leg pair, and the direction of forces acting on them.

Both spider species differed in the relative use of their third and fourth legs for the jump. *P. lanigera* had their third legs in contact during the whole acceleration phase, whereas their fourth legs detached earlier. In contrast, in *S. pubescens*, the fourth legs were in contact throughout the acceleration phase, and the third legs detached earlier. It has been shown that the relative contribution of third and fourth legs varies between different species of jumping spiders, and is a function of the ratio of third to fourth leg length (Ehlers 1939; Parry and Brown 1959a; Hill 2018). Consistently, we found a much greater relative length of third legs in *P. lanigera* than in *S. pubescens* (length ratio 0.92 vs. 0.69). A quantitative analysis of the relative contributions of third and fourth legs would require measurement of their individual ground reaction forces, but the longer contact duration, and the larger adhesive contact area of the claw tufts confirm the dominance of third legs in jumps of *P. lanigera* and of fourth legs in *S. pubescens*.

The use of third and fourth legs for jumping also has important implications for the foot contact and the use of the pretarsus during the acceleration. Most importantly, the tip of the third leg is pointing forward, and *pulled* backwards to accelerate, whereas the fourth leg tip is pointing backwards and *pushed* backwards to accelerate. We found that the pretarsi of the legs mainly powering the jump (third in *P. lanigera*, fourth in *S. pubescens*) were more aligned with the axis of the jump than the pretarsi of the other leg pair, and became even more aligned during the acceleration phase.

The different direction of forces (pushing vs. pulling) in the third and fourth legs has consequences for the contact of claw tuft setae. We found that only some setae in different parts of the claw tuft came into close surface contact during the acceleration phase, namely setae on the lateral or distal side of the claw tuft in the pulling third legs, and setae on the proximal side of the claw tuft in the pushing fourth legs. A similar specialization of proximal and distal parts of the tarsus and pretarsus for pushing and pulling has been shown for many climbing insects. While distal adhesive pads stick firmly when pulled toward the body (but detach when pushed), the proximal friction pads allow legs to push without slipping (Clemente and Federle 2008; Bullock and Federle 2009; Labonte and Federle 2013).

Direction-dependence and specialization for pushing and pulling has also been reported for spiders which possess tarsal scopulae and pretarsal claw tufts. Scopula hairs have microtrichia-covered sides facing in the distal direction of the leg, whereas setae with microtrichia-covered sides facing in the proximal direction are found on the pretarsal claw tuft (Niederegger and Gorb 2006; Wolff and Gorb 2013). Force measurements on scopula hairs in *Aphonopelma seemanni* (Theraphosidae) and *Cupiennius salei* (Ctenidae) confirmed that they are anisotropic and produce higher shear forces in the pushing direction (Niederegger and Gorb 2006). Moreover, it has been shown for bird spiders that during locomotion, tarsal scopula hairs are used for pushing, whereas pretarsal claw tufts are used for pulling and adhesion (Pérez-Miles et al. 2015).

While tarsal scopula setae are present in some salticids (Hill 2010c), they are absent in the third and fourth legs of *P. lanigera* and *S. pubescens*, and the close contacts we observed during the acceleration phase of jumps were made by claw tuft setae. Given that in the species studied so far claw tuft setae mainly point in the distal direction, how can the claw tufts of fourth legs produce pushing forces, in particular in *S. pubescens* where mainly the fourth legs produce the jump?

**Fig. 6:**
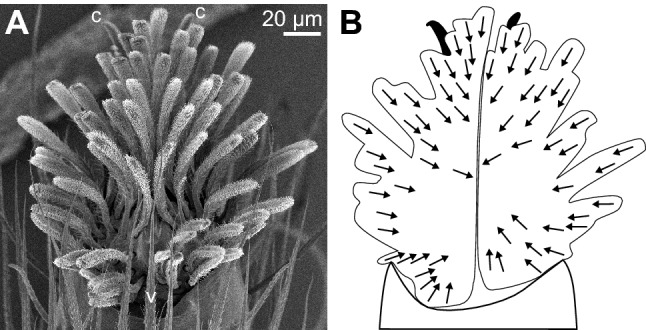
SEM image of fourth leg pretarsus of *S. pubescens* (**a**) and diagram of the same pretarsus with arrows indicating the orientation of the microtrichia-covered side of the setae

Our SEM observations show that the orientation of many claw tuft setae in *P. lanigera* and *S. pubescens* deviates significantly from a distal direction. In particular, setae on the lateral margins of the claw tufts are pointing laterally, and their microtrichia-covered sides are facing inward (Figs. 5, 6). Effects of such a lateral orientation of setae have been documented for the adhesive hairs of *Gastrophysa viridula* leaf beetles and *C. salei* hunting spiders (Bullock and Federle 2009; Wolff and Gorb 2013). In *G. viridula*, the middle and proximal tarsal pads have more laterally oriented hairs, allowing them to support higher lateral friction forces than the distal pads (Bullock and Federle 2009). When pads are sheared laterally, the setae on the side opposite to the sliding direction can produce high friction forces. In *C. salei*, the distal and lateral orientation of claw tuft adhesive setae, and the proximal orientation of tarsal scopula setae may allow the spiders to resist shear forces in different directions.

Interestingly, the claw tufts in the fourth legs of *S. pubescens* show a special radial arrangement of the setae which differs both from their own third legs and the fourth legs of *P. lanigera* (Figs. 5, 6). This radial seta arrangement may allow the legs to resist shear forces in all directions, analogous to the digits of climbing tree frogs and geckos, which are also spread radially so that their toe pads can resist shear forces in multiple directions. Moreover, vertically climbing frogs and geckos can actively adjust the orientation of their limbs and digits for head-up, head-down or lateral climbing, so that always some toes are pointing upwards, in the correct orientation to support the body weight by pulling (Hanna and Barnes 1991; Birn-Jeffery and Higham 2014; Russell and Oetelaar 2016).

The spiders’ claw tuft setae can also re-orientate, but here the movement is passive. When the pull of a leg is directed towards the base of adhesive setae (e.g. a proximal pull when hairs are oriented in the distal direction), the setae are under tensile stress, stabilizing their orientation. However, if the pull deviates from this direction, two outcomes are possible: first, if the legs are pushing towards the tips of adhesive setae, the hairs will buckle and their adhesive tips may detach or flip over and touch the surface with their backside which is not adhesive (Bullock and Federle 2009; Wolff and Gorb 2013); second, if the pull on the legs deviates by a smaller angle from the direction towards the hair base, the setae can re-orientate and align themselves to the pull while still remaining in adhesive contact with the surface. Reorientation of distally directed hairs in response to pushing forces has been described for the tarsal friction hairs of ants (Endlein and Federle 2015).

The direction-dependence of setae is highly beneficial for the extremely rapid switch between attachment and detachment required for successful jumping. The lateral and distal claw tuft setae that are attached when the third leg moves backwards during the acceleration phase will easily detach when the leg is moved forward at take-off. Similarly, the setae on the proximal claw tuft of the fourth legs will easily detach at take-off.

The unusual seta orientation of the claw tufts in the fourth legs of *S. pubescens*, and their greater length along the proximal–distal axis, are likely adaptations for jumping from smooth surfaces, allowing the long fourth legs to produce strong pushing forces. Further studies need to clarify whether this claw tuft morphology is more common among salticids, and indeed mainly present in species which propel their jumps with the fourth legs.

In comparison to some of the most powerful jumping insects of similar size, the jumps of salticid spiders including *P. lanigera* and *S. pubescens* are slow [best jumps of froghoppers and planthoppers: 4.7 m/s and 5.8 m/s (Burrows 2006, 2014); salticid jumps: 0.4–1 m/s (Hill 2018)]. Therefore, the estimated power per muscle mass is relatively small, of the order 20 W/kg, allowing salticids to power their jumps by muscle contractions, as opposed to stored energy via a catapult mechanism (Nabawy et al. 2018).

Assuming that jumping spiders accelerate uniformly from the onset of the movement of third and fourth legs until take-off, we can estimate the shear forces acting on the legs in contact as $F=mv\mathrm{cos}\alpha /t$, where $m$ is the body mass, $v$ is the take-off velocity, $\alpha$ is the take-off angle and $t$ is the acceleration time. From the mean values for *P. lanigera* and *S. pubescens*, the shear total shear force is 0.16 mN for *P. lanigera* and 0.55 mN for *S. pubescens*. Assuming that only one dominant leg pair is accelerating, and that the force per claw tuft acts on the measured peak contact area, a shear stress of 0.4 MPa (*P. lanigera*) and 1.1 MPa (*S. pubescens*) is obtained. This shear stress is lower than the measured force per projected contact area of single gecko setae (4.6 MPa; Autumn and Puthoff 2016). It is likely that the spiders’ claw tufts would also generate sufficient friction on micro-rough surfaces, where the adhesion of claw tufts is only slightly reduced (Wolff and Gorb 2012).

The low take-off velocity also means that the forces on the substrate produced by jumping spiders’ legs are small. While froghoppers can produce forces as high as 33 mN per leg and use this force to pierce plant surfaces with their sharp tarsal spines (Burrows 2006, Goetzke et al. 2019), the forces for salticids are two orders of magnitude smaller, and may therefore make this type of gripping mechanism impossible. At the same time, small forces may be favourable for the use of soft attachment structures such as claw tufts, as they are exposed to less wear and damage.

We observed that the erection of leg spines before a jump took place only 0.5 ms earlier in the third than in the fourth legs, and shortly after the first movement became visible (Table 1). This pattern suggests that the hemolymph pressure increases almost simultaneously in all legs before a jump. While the spine erection during jumping has been interpreted as a side effect of the hydraulic extension of the propulsion legs (Parry and Brown 1959b), it might also play a role in facilitating locomotion across challenging substrates containing gaps or holes: as the spines are spread out during leg extension, they are more likely to catch on protrusions of the substrate; they can transmit high forces to the substrate due to their high stiffness when bent away from the leg. On the other hand, the spines collapse during leg flexion, and when they are pushed toward the leg segment; this will allow easy leg pull-out of the leg without entanglement (Spagna et al. 2007).

It has been shown for the jumping spider *Euophrys frontalis* that increased hemolymph pressure can not only erect the leg spines, but also spread the claw tufts in the lateral direction (Wolff and Gorb 2016). However, we did not observe any lateral spreading of the claw tufts when the leg spines were erected. This suggests that at least for the two species of jumping spiders studied here, the claw tufts might require higher pressures to spread laterally, and these were not reached during the acceleration phase of the jump. The contact area changes we observed in the third and fourth legs during the acceleration phase were small and not based on a lateral spreading of the claw tuft, but on the closer contact of individual setae.

The jumps of salticid spiders are highly controlled and targeted, and they do not show any uncontrolled spin that is observed in many jumping insects (Burrows 2010, 2012; Clemente et al. 2017). Because of their footfall position anterior to the body centre of mass, a forward and upward acceleration produced by the third legs will always result in a backward pitching moment on the body (counterclockwise in Fig. 1, tending to lift the head). In addition to the pitch stabilization by the dragline silk thread (Chen et al. 2013), it is likely that this backward pitching moment is also compensated by a forward pitching moment from the fourth legs. The use of two legs for jumping may not only facilitate the control of body pitch but also help to further reduce forces on the soft adhesive structures (Burrows 2011).

The spiders’ forward jumps with small take-off angles observed in this study imply that their legs can produce friction forces significantly larger than the normal load, corresponding to a large friction coefficient (> 1). The spiders achieve this high friction coefficient by the contact of their adhesive claw tufts, which strengthen the contact with the substrate during the acceleration phase. The essential role of the claw tufts for jumps from smooth surfaces is confirmed by our claw tuft ablation experiments on the third or fourth legs, which caused the manipulated legs to slip when the spiders attempted to jump. High friction coefficients enable jumping spiders to perform controlled and targeted jumps independent of the substrate surface (smooth, rough) and orientation. This is crucial for moving in challenging terrain and successfully catching prey.

The adaptations of spiders for jumping from smooth surfaces reported here provide a further striking example of very rapid control of surface attachment in arthropods.

## Supplementary Information

Below is the link to the electronic supplementary material.

Supplementary file1 (PDF 248 KB)

Supplementary file2 (PNG 188 KB)

Supplementary file3 (MP4 4515 KB)

Supplementary file4 (MP4 18436 KB)

Supplementary file5 (MP4 3438 KB)
