# Supplementary material for: Role of legs and foot adhesion in salticid spiders jumping from smooth surfaces
Source: J Comp Physiol A Neuroethol Sens Neural Behav Physiol. 2021 Mar 10;207(2):165–77. doi: 10.1007/s00359-021-01466-6 (PMC8046696; doi:10.1007/s00359-021-01466-6)
Supplement: Supplementary file 1 — Supplementary file1 (PDF 248 KB) [file 359_2021_1466_MOESM1_ESM.pdf]

Supplementary Material

Role of legs and foot adhesion in salticid spiders jumping from smooth surfaces

Hanns Hagen Goetzke[1], Walter Federle[2]*

University of Cambridge, Department of Zoology, Cambridge, United Kingdom

*corresponding author: wf222@cam.ac.uk

[1]ORCID: 0000-0002-9204-278X

[2]ORCID: 0000-0002-6375-3005

Supplementary Video 1:

Pseudeuophrys lanigera jumping from glass. Side view of the jump, and magnified views of third and fourth leg from below using bright field epi-illumination.

Supplementary Video 2:

Sitticus pubescens jumping from glass. Side view of the jump, and magnified views of third and fourth leg from below using bright field epi-illumination.

Supplementary Video 3:

Jumps with contaminated claw tufts, or experimental ablation of setae.
a) Failed jump of *Pseudeuophrys lanigera*, caused by slipping of contaminated third legs;
b) jump of *Sitticus pubescens*, with claw tuft setae of third legs ablated;
c) jump of *Sitticus pubescens* with claw tuft setae of left fourth leg ablated.
Despite the slipping of the manipulated legs, *S. pubescens* spiders were still able to perform controlled jumps towards the target.

Measurement of take-off angle and take-off velocity

Take-off angle α and take-off velocity v were calculated from the jump trajectory seen through camera 1 and 2 (Fig. S1). The jump direction ϑ relative to camera 2 can be measured from the ventral view (inverted microscope, a forward jump and onto the fly would be ϑ = 90°), and a projected take-off angle γ as well as the velocity components in camera 2 $\acute{v}_x$ and $\acute{v}_y$ in the

horizontal and vertical camera direction can be measured from the side view. Having measured the angle β between camera 2 and the glass coverslip, the take-off angle α can be calculated as

$$\alpha = tan^{-1}(cos(\vartheta - 90°)tan\gamma / cos\beta)$$

The take-off velocity v along the direction of the jump was calculated as

$$v = \sqrt{(\acute{v}_x / sin\vartheta)^2 + (\acute{v}_y / cos\beta)^2}$$

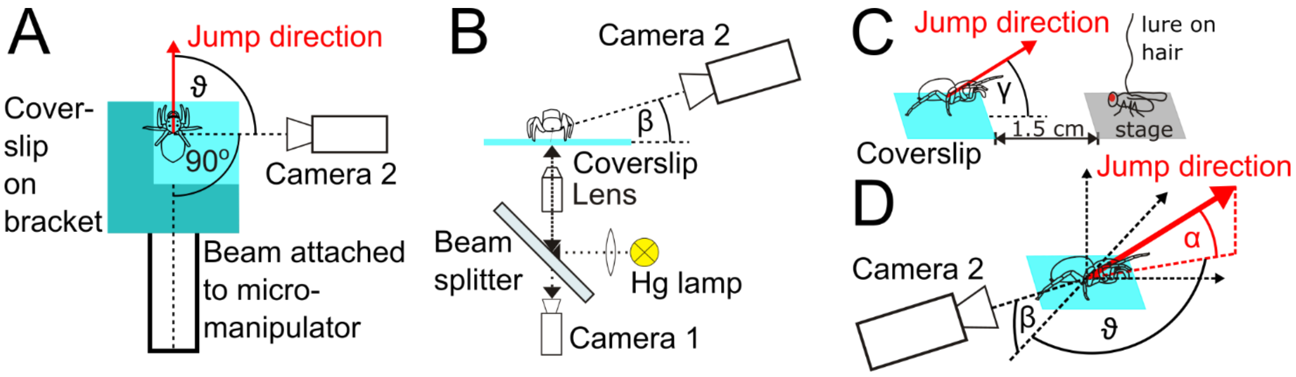

Figure S1 Sketch of the angles measured to calculate the take-off angle α in jumps from the inverted microscope. (A) View from above: the jump direction ϑ can be determined from the contact area recordings filmed by camera 1 from below. (B) View from the side: camera 2 is oriented to the surface at angle β to allow recordings of the tarsi. (C) View from camera 2: a projected take-off angle γ can be determined from the video. (D) The real take-off angle α can be calculated using ϑ, β, and γ.
