# Supplementary figures and images for: Role of legs and foot adhesion in salticid spiders jumping from smooth surfaces
Source: J Comp Physiol A Neuroethol Sens Neural Behav Physiol. 2021 Mar 10;207(2):165–77. doi: 10.1007/s00359-021-01466-6 (PMC8046696; doi:10.1007/s00359-021-01466-6)

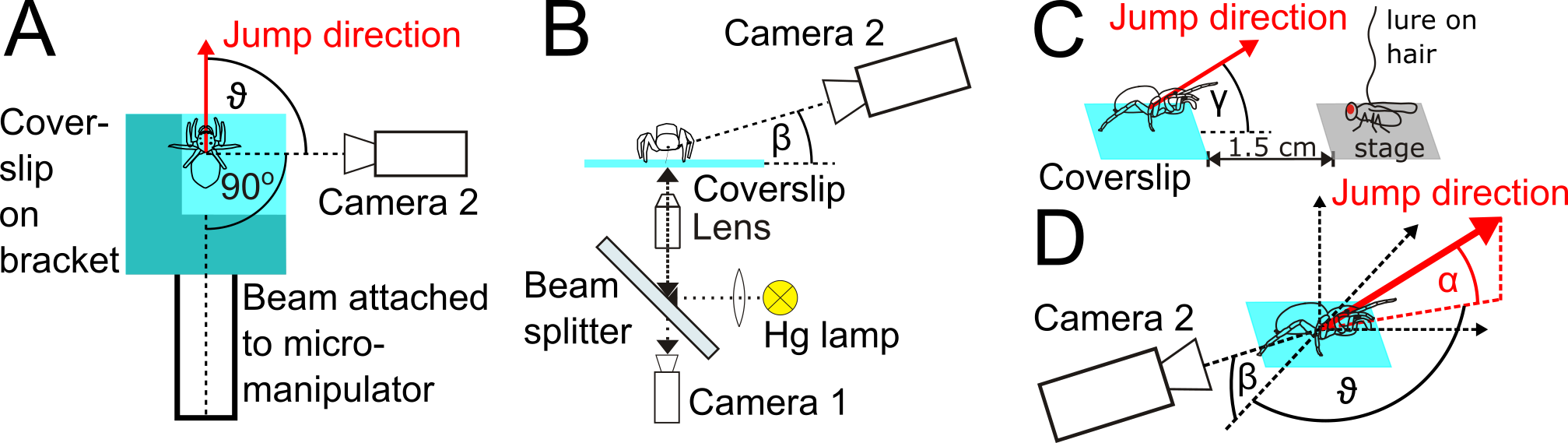

Supplement: Supplementary file 2 — Supplementary file2 (PNG 188 KB) [file 359_2021_1466_MOESM2_ESM.png]
